# Nonclinical regulatory considerations for investigational new drug applications for regulatory T cell therapies

**DOI:** 10.3389/fmed.2025.1626067

**Published:** 2025-11-24

**Authors:** Phuong-Uyen Dinh, Lauren Mihalcik

**Affiliations:** Aclairo Pharmaceutical Development Group, Sterling, VA, United States

**Keywords:** Treg, FDA, nonclinical, CAR, guidelines, toxicology, pharmacology, drug development

## Abstract

Since FDA approval of the first chimeric antigen receptor (CAR) T cell therapy in 2017, the landscape of cell-based therapies has widely expanded. This expansion has encompassed both the types of cell therapies being developed as well as indications beyond oncology. As an example, the number of regulatory T cell (Treg) therapies in development have been steadily increasing, with targeted focus on treatment of autoimmune and inflammatory diseases. The nonclinical development pathway for Treg therapies has relied on leveraging existing regulatory guidance documents for cell and gene therapies, however the lack of Treg specific guidance coupled with often times limited appropriate preclinical models have created regulatory challenges for drug developers. In this review, preclinical considerations for the development of Treg therapies will be described. Specifically, topics will include the following: (1) Current health authority expectations for demonstrating pharmacodynamics, biodistribution, and safety of Treg therapies, including selection of relevant models, assay selection for bioanalytical endpoints, and strategic study planning to maximize study readouts while reducing animal use. (2) Approaches for conducting target liability assessments to supplement nonclinical development packages for indications with limited appropriate preclinical models. (3) Leveraging health authority interactions, such as the US Food and Drug Administration (FDA) Initial Targeted Engagement for Regulatory Advice on CBER/CDER Products (INTERACT) and Pre-Investigational New Drug (Pre-IND) meetings, to gain feedback and actionable directives to guide program development. (4) Guidance on generating high quality nonclinical regulatory documents, including study reports and submission documents, to support IND applications.

## 1 Introduction

Regulatory T cells (Treg) are a subset of T cells that play a crucial role in maintaining immune homeostasis by suppressing excessive immune responses, preventing autoimmunity, and controlling inflammation (1–3). Deficiency of Treg function can lead to the development of autoimmune diseases and other immune-mediated disorders such as Type 1 diabetes, rheumatoid arthritis, and multiple sclerosis (4–6). Therefore, Treg-based immunotherapies that harness the power of T cells to fight autoimmune diseases holds great promise. However, the development pathway for such therapies to achieve regulatory approval is rigorous and multi-faceted.

Since FDA approval of the first chimeric antigen receptor (CAR) T cell therapy in 2017 (7, 8), the landscape of cell-based therapies has widely expanded (9, 10, 31). This expansion has encompassed both the types of cell therapies being developed as well as indications beyond oncology. The number of Treg therapies in development have been steadily increasing, with targeted focus on treatment of autoimmune and inflammatory diseases (11, 12). These therapies can be autologous or allogeneic and include CAR-Treg which are activated in the presence of a specific ligand and T-cell receptor (TCR)-Treg therapies which are activated in the presence of a peptide presented by major histocompatibility complex (MHC). The nonclinical development pathway for Treg therapies has relied on leveraging existing regulatory guidance documents for traditional biologics and CAR-T (13); however, the lack of Treg specific guidance coupled with often times limited appropriate preclinical models have created regulatory challenges for drug developers.

The development of Treg therapies, similar to CAR-T-cell based therapies, requires careful consideration of nonclinical safety and efficacy. These assessments include on- and off-target toxicity, cytokine release syndrome and neurotoxicity, gene editing and vector integration, efficacy evaluation, manufacturing and quality controls, and regulatory strategies. Treg therapy holds great promise for treating immune-mediated diseases by restoring immune balance. In essence, the regulatory considerations are to ensure that Treg therapies are safe, pure, potent, and effective, before they can be used in human clinical trials by leveraging (1) current health authority expectations for demonstrating pharmacodynamics, biodistribution, and safety; (2) target liability assessments to supplement nonclinical development packages for indications with limited appropriate preclinical models; (3) appropriate health authority interactions to gain feedback and actionable directives to guide program development; and (4) guidance on generating high quality nonclinical regulatory documents to support investigational new drug applications (INDs) applications.

## 2 Review of applicable guidelines for the development of regulatory T cell therapies

Developing cell therapies, like CAR-Treg or T cell receptor (TCR)-Treg therapies, requires careful navigation of the regulatory landscape and adhering to international and local guidelines. While the International Council of Harmonisation (ICH) aims for global harmonization, the various health authorities have their specific regulations and guidance documents tailored to their respective regions. Sponsors need to consider these guidelines throughout the development process, from preclinical studies to clinical trial and post-market surveillance, to ensure the quality, safety, and efficacy of these innovative therapies in patients across different regions.

While there are no specific ICH guidelines solely dedicated to cell therapies, several existing guidance documents contain relevant information including Q1A (R2) (35), Q5B (36), Q5D (37), S6 (R1) (38) and S12 (24). Regionally, the Food and Drug Administration (FDA) and European Medicines Agency (EMA) have published some key regulatory considerations and guidance for cell and gene therapies in general as well as some specific to indications and cell source or cell types. Some key guidance documents and their relevance to development of cell therapies are presented in Table 1.

**Table 1:** Applicable guidelines for T cell therapies.

Function	Guidance	Relevance
General	ICH S6 (R1) (38)	Provides the framework for the preclinical safety evaluation of biologics including cell therapies, which outlines guidance on CMC, species selection, preclinical safety evaluation, and pharmacokinetics and pharmacodynamics assessment.
	Guidance for Industry: Considerations for the Development of Chimeric Antigen Receptor (CAR) T Cell Products (January 2024) (15)	Provides specific recommendations for CAR-T development regarding CMC, pharmacology and toxicology, and clinical study design.
	Guidance for Industry: Human Gene Therapy Products Incorporating Human Genome Editing (January 2024) (27)	Provides general considerations for products with genome editing or modifications and specific recommendations for CMC, pharmacology and toxicology, and clinical study design.
	Regulation (EC) No 1394/2007 of the European Parliament and of the Council of 13 November 2007 on Advanced Therapy Medicinal Products (December 2007) (14)	Provides guidelines on the quality (CMC), nonclinical, and clinical aspects of cell and gene therapy products including genetically modified cells (CAR-T), human cell-based products including stem cells and non-genetically modified cells.
CMC	ICH Q1A (R2) (35)	Primarily for small and large molecules, the general principles of stability testing outlines may still be relevant.
	ICH Q5B (36)	Provides guidelines for analysis and characterization of recombinant DNA derived products such as CAR-T and modified Tregs.
	ICH Q5D (37)	Provides guidance on the source, history, and characterization of cell lines, including cell banks, used in manufacturing.
	Guidance for Industry: Chemistry, Manufacturing, and Control (CMC) Information for Human Gene Therapy Investigational New Drug Applications (INDs) (January 2020) (33)	Provides recommendations regarding CMC for gene therapies and cell-based gene therapies.
Pharmacology	Guidance for Industry: Potency Tests for Cellular and Gene Therapy Products (January 2011) (32)	Provides recommendations regarding potency measurements, potency assay designs and validations for cell and gene therapies. Building upon the January 2011 guidance on potency test, the 2023 draft guidance provides additional recommendations regarding acceptance criteria using a science- and risk-based approach.
	Draft Guidance for Industry: Potency Assurance for Cellular and Gene Therapy Products (December 2023) (34)	
Pharmacokinetics	ICH S12 (24)	Primarily focused on gene therapy, but the principles of biodistribution assessment are relevant for understanding the fate of cell therapies *in vivo*.
Toxicology	Guidance for Industry: Preclinical Assessment of Investigational Cellular and Gene Therapy Products (November 2013) (13)	Provides specific recommendations for species selection, study design, safety endpoints and assessment of cell survival, engraftment, biodistribution, and cell fate for cell therapies.

For Advanced Therapy Medicinal Products (ATMP) like cell and gene therapies, both the FDA and EMA recommend a science- and risk-based approach and encourage early discussion with regulatory authorities (13–15). While there are guidances on ATMP classification, manufacturing and product testing requirements, nonclinical safety and efficacy guidances are largely based on following a risk-based approach taking into consideration the specific characteristics of the product (cell type, biologic source, and manufacturing process), mechanism of action, and intended use. Unlike traditional small and large molecule products, these novel therapies also possess potential long-term risk such as clonal expansion and transformation of the infused cells, therefore, both FDA and EMA emphasize the importance of long-term follow-up due to any potential delayed adverse events (16–18).

Besides S12, there is no other cell and gene therapy specific guidance from the ICH. However, the ICH has recognized the unique challenges and complexity of the nonclinical, clinical, and manufacturing of ATMPs that is not fully addressed in the current ICH guidelines for traditional biologics. Coupled with the rapid advancements in ATMPs and the growing number of therapies in the clinic, ICH recognizes the need for a more strategic framework to address the harmonization needs for ATMPs. This has led to the formation of the Cell and Gene Therapy Discussion Group (CGT DG), which plans to release a holistic CGT roadmap and a recommendation paper summarizing high level principles and global regulatory framework which will cover both *in vivo* viral vector-based gene therapy products as well as *ex vivo* genetically modified cells, such as CAR-T and Treg products (19).

## 3 Regulatory expectations for nonclinical development packages

### 3.1 Demonstration of efficacy

Demonstrating efficacy of human Treg cell therapy products in the nonclinical context is challenging due to the complex nature of the products and requirement for disease models with competent immune systems amenable to human Treg treatment. For many human-specific therapeutic products, a species-specific surrogate could be considered, but this approach has proven to be difficult due to the complexity of Treg products. Optimized CARs or TCRs with multiple components, including IL-2 support components, gene edits to lock in phenotype, expression tags, or kill switches, incorporated into these products make designing an equivalent surrogate product difficult (20–22). Each component has the potential to have a species-specific issue, whether it is lack of recognition of endogenous signaling molecules or incompatibility with detection reagents.

Even for a relatively simple CAR Treg with no other components, creating a surrogate mouse CAR Treg which can be used in a mouse model of disease is complicated by factors of species specificity for the target and by the difficulties associated with harvesting enough Treg cells from mice to produce therapeutic quantities of the surrogate products to both characterize and use *in vivo*. For these reasons and the uncertainty of clinical translation, surrogate cell models, whether using mice or larger species, are not widely used in the authors’ experience.

Because of the technical limitation of *in vivo* models, these kinds of experiments are typically not the key pieces of data to support efficacy of Treg cell products. In the authors’ experience, *in vivo* efficacy studies, if conducted, are typically limited to graft vs. host disease (GvHD) models in immunodeficient mice transplanted with human peripheral blood mononuclear cells, where the system can show general anti-inflammatory effects of the Treg cells in the context of a human immune system. These models often do not incorporate the ligands for the CAR or TCR, making the *in vitro* experiments of primary importance.

*In vitro* experiments in which the Treg product disrupts a disease-relevant inflammatory process or interaction are the most important evidence of efficacy typically provided. These *in vitro* studies, combined with literature-based arguments around the importance of the CAR or TCR target to the disease process and general scientific knowledge about Treg cells, can provide a scientific rationale for efficacy and a justification for conducting a clinical trial in patients.

*In vitro* experiments required to support clinical trials are highly product dependent. These can include:

- Demonstration of the dose–response for activation by the CAR or TCR target
- Characterization of the cytokine response to activation to demonstrate that the Tregs produce (e.g.) IL-10 and TGFβ rather than an effector T cell cytokine profile
- *In vitro* efficacy studies using patient cells and disease-relevant biomarkers
- Characterization of the stability of the Treg phenotype over time and with repeated stimulation
- Characterization of any kill switches using appropriate stimulations
- Studies which characterize and justify the use of other components of the product (e.g., IL-2 support)
- For clinical trials in pediatric populations, additional efficacy studies may be required to show evidence of the prospect for direct benefit (23)

### 3.2 Off-target characterization

There is an expectation that the potential for off-target binding of a CAR, modified TCR, or other targeting ligand will be evaluated in an appropriate assay or series of assays. These could include tissue cross-reactivity assays using a fusion protein with the recognition domain, protein or cellular arrays, or experiments to identify the specificity for peptide-HLA combinations for TCR-Treg. In addition to endogenous human off-targets, the potential of Treg products to inhibit needed responses to foreign antigens should be considered.

### 3.3 Biodistribution

For some gene therapy products like an AAV or gene editing products, biodistribution studies are frequently conducted, but are generally not appropriate for *ex vivo* modified Treg products as widespread distribution of these products is expected (24). Evaluation of distribution of Treg products to specific tissues can however be used to aid in interpretation of toxicology findings, to confirm distribution to a specific target organ (e.g., across the blood brain barrier for neurological indications), or to justify a route of administration. If conducted, these studies are typically performed in immunocompromised animals using the clinical Treg drug product.

### 3.4 Safety

As with most cell and gene therapy products, there is not a standard roadmap to evaluate the safety of an investigational Treg product to support opening clinical trials. Safety assessments for Treg products share some characteristics with effector T cell products [described in a review based on a Health and Environmental Sciences Institute (HESI)/FDA workshop, (25)]; however, the unique properties of Treg cells (being generally anti-inflammatory rather than pro-inflammatory or cytotoxic) imply different risks than effector T cells. Nonclinical safety programs should be designed to evaluate the specific risks associated with the product, considering the safety of administration of autologous unmodified Treg products (11, 26). Major potential risks for Treg products include uncontrolled proliferation (potentially leading to oncogenicity), overactive immunosuppression, sequalae of loss of Treg phenotype (i.e., conversion to conventional T cells), and immune rejection, particularly of allogenic products. There may be additional major potential risks identified based on the product characteristics.

#### 3.4.1 Target liability assessments

The safety assessment for a new Treg product starts with a comprehensive literature review to ascertain what is known about cross-tissue expression of the CAR or TCR target. For CAR-Treg, this can be reasonably straightforward as there is extensive information on protein and mRNA expression in publicly available sources such as the Human Protein Atlas. For TCR-Treg, this analysis can be similarly conducted but with the additional context that targets expressed in a tissue may not be displayed in an appropriate context to activate the drug product. If sufficient information exists in public databases to characterize potential target organs, additional expression work may not be needed. If there is not sufficient information available for the target, additional work to look at either mRNA or protein expression across major organs in humans may be needed.

Based on the potential target tissues identified by expression analysis and a comprehensive literature review, potential risks can be identified which guide the selection of experiments with the drug product or surrogate products.

#### 3.4.2 *In vitro* safety assessments

Because traditional toxicology studies are challenging for human cell products due to development of graft vs. host disease, *in vitro* safety assessments frequently provide the bulk of the safety information for Treg products. The specific experiments vary based on the product characteristics, but in the authors’ experience, these assays can include:

- Cytotoxicity assays to demonstrate a lack of cell killing in cell lines representing potential target tissues (e.g., cell lines derived from tissues where the target is expressed)
- An assessment of cytokine (IL-2) independent growth as a potential assessment of uncontrolled proliferation
- Evaluations to identify whether there are dominant clones
- Evaluations to characterize the safety of additional components or transgenes
- Evaluations to characterize the safety of the product in the context of co-administered drugs or biologics

#### 3.4.3 *In vivo* safety assessments

While *in vivo* toxicology studies are key components of the safety assessment for traditional therapeutics, it can be challenging to identify a relevant *in vivo* model for safety assessments of human cell therapies. Short-term *in vivo* studies in immunodeficient rodents can provide some assurance of safety and *in vivo* stability, but even these models are limited by development of graft vs. host disease. In the author’s experience, most IND-enabling programs for Treg therapies include such studies using the human drug product with standard histopathology assessments and evaluation of Treg persistence. Species-specific surrogate products could also theoretically be useful to evaluate the safety of the overall approach, but these products can be very challenging to design and produce in sufficient quantities to conduct safety assessments.

#### 3.4.4 Genetic toxicity

The extent of genetic toxicity evaluation needed for Treg products depends on the method used to produce them. For products produced with modern integrating viral vectors (e.g., lentivirus), the genetic toxicity assessment can often be limited to an integration site analysis to confirm integrations are not preferentially occurring near potential oncogenes, assuming there is no evidence of a dominant clone from CMC assessments. For products made using gene editing, a more extensive assessment is required (27, 28).

#### 3.4.5 Immunogenicity

There is no standard method for evaluating the potential immunogenicity of a Treg product, as the risks of immunogenicity will vary depending on the specific product characteristics. *In vivo* assessment of immunogenicity (e.g., measuring anti-CAR antibodies) can sometimes be useful to understand and explain observed toxicities *in vivo*, but these assessments are unlikely to be useful for predicting human immunogenicity because of the use of humanized proteins in a nonclinical species. For some programs, *in vitro* assessments of immunogenicity using human cells could be helpful for rank ordering potential therapeutic candidates. The risks associated with potential immunogenicity of product components should be assessed prior to clinical trial initiation so that appropriate clinical monitoring and mitigation strategies can be included in the clinical trial design.

## 4 Regulatory documentation

For INDs submitted to the FDA, guidelines for the kinds of data which should be submitted are described in the FDA’s Guidance for Industry: Preclinical Assessment of Investigational Cellular and Gene Therapy Products (13). The main source of data evaluated for INDs are the individual study reports submitted in Module 4 of the eCTD. Additionally, sponsors frequently submit written and tabulated summaries of the nonclinical data (found in section 2.6 of the eCTD) and a nonclinical overview (found in section 2.4), discussed in more detail below.

### 4.1 Study reports

Study reports are arguably the most important documents in an IND dossier for any drug product. Having high quality, well-written, and thorough reporting of the experiments conducted increases trust with reviewers and shows a sponsor commitment to quality and transparency. A report should be submitted for each *in vitro* or *in vivo* nonclinical study that supports the safety of the product. Complete pharmacology reports are also frequently submitted but are not required so long as sufficient pharmacology data is included in written summaries to allow an independent interpretation of the results (13). Reports for safety-related studies should include a prospective protocol (with amendments), details of the study design, a complete set of results, including individual animal data, and an interpretation of the results.

For some studies, electronic datasets in the Standard for Exchange of Nonclinical Data (SEND) format are required. SEND requirements are described in the FDA Data Standards Catalogue (29), which lists the relevant data standards and the dates that the datasets become required. Whether SEND is required for a study depends on several factors:

- The type of study: at the time of publication, Center for Biologics Evaluation and Research (CBER) requires SEND datasets for single- and repeat-dose toxicology studies (filed in Module 4.2.3.1 or 4.2.3.2) and carcinogenicity studies (filed in Module 4.2.3.4) (if conducted) and *in vivo* genetic toxicology studies (filed in Module 4.2.3.3.2). Any study in which both pharmacology and toxicology endpoints are evaluated would be required to have SEND datasets if the primary purpose of the study was safety.
- The date the study was conducted: Studies which predate the beginning of the requirement for SEND are excluded from the requirement. For each study type, the data standards catalog provides a start date for the requirement. If the study was started (i.e., the protocol was signed by the study director) before the date the requirement begins, the SEND datasets would not be required. For general toxicity studies submitted to CBER, studies initiated prior to 15 March 2023 do not need SEND datasets. For *in vivo* genetic toxicity studies, the requirement begins on 15 March 2025 (29).

Note that specific SEND requirements differ between CBER and Center for Drug Evaluation and Research (CDER).

### 4.2 Nonclinical summary documents

Nonclinical summary documents filed with an IND are important for providing an overall justification of the nonclinical strategy. This is particularly true for products regulated by CBER, which have fewer “checkboxes” than traditional small molecule programs and often require unique collections of experiments to evaluate safety and efficacy. A full description of the nonclinical summary sections is beyond the scope of this review, but specifically for Treg programs, special attention should be paid to the description and justification of models used in the development program. Key points to cover [either in the Overview of the Nonclinical Testing Strategy in Module 2.4 or in the Pharmacology Written Summary (2.6.2)] to be sure the reviewers understand how the package fits together include:

- Discussion of the previous use of unmodified Treg in the indication
- Justification of any efficacy models and how they relate to the human condition in terms of disease severity, onset, and reversibility
- Strengths and weaknesses of the models used
- Justification of the ages of the animals used (if relevant)
- Justification for limited *in vivo* testing
- Comparison of the key quality attributes of the lots of material used in nonclinical studies, noting which are representative of the clinical material
- Full descriptions of any surrogate products used, ideally with a table describing how they are similar and different to the clinical drug product
- A detailed risk assessment with discussions of potential mitigations

Outside of these sections justifying the nonclinical testing strategy, submission processes tend to go most smoothly (in the authors’ experience) when the nonclinical summaries contain very little that is not also in a nonclinical study report. If there are additional cross-study analyses that need to be done to define pharmacokinetics (PK)/ pharmacodynamics (PD) relationships or justify cross-species comparisons, it can be very helpful to include these as separate, quality controlled, study reports rather than only having these analyses in the summary documents.

## 5 Importance of health authority interactions

Staying up to date on the evolving regulatory expectations and engaging in early discussions with regulatory bodies are crucial for efficient product development and approval. For nonclinical development questions, INTERACT and pre-IND meetings provide opportunities to ask questions about Agency expectations for a specific program.

INTERACT meetings ideally occur earlier in development, when there is a specific development candidate identified and some preliminary proof of concept data is available. These meetings are ideal for gaining alignment on the *in vitro* and *in vivo* models to be used in the program and the overall nonclinical strategy. General information about these meetings is available in the INTERACT SOPP 8101.1 (30). Questions about the design of pivotal safety assessments, aside from the model to be used, can trigger denial of a meeting as premature as they are generally considered outside the scope of INTERACT meetings.

Pre-IND meetings ideally occur when there is enough pharmacology data available to support identification of a minimally efficacious dose and preliminary toxicology data to support dose selection for the pivotal safety evaluation. Regulatory feedback from CBER consistently requests that Sponsors have their pre-IND meetings prior to the start of the pivotal toxicology studies.

In preparation for these meetings, the quality of the information provided in the briefing books determines how useful the regulatory feedback will be. Sponsors should think carefully about what data reviewers would need to answer the questions they propose in a meaningful way. For nonclinical questions, this often includes information from CMC and clinical disciplines (e.g., impurity information across lots used in nonclinical studies or the age or reproductive status of the intended patient population).

## 6 Nonclinical considerations for clinical trial dose selection

Because Treg products are designed to proliferate *in vivo*, the clinical starting dose is typically not dictated by the results of animal studies. The results of clinical studies with similar or analogous products are considered more relevant, and doses may be modified based on the results of characterization studies (15). For these products, calculating a starting dose from an animal NOAEL and a safety margin, as would typically be done with a small molecule, is generally not appropriate.
